# Effects of Pilates Training on Cardiorespiratory Functions in Medical Conditions - Comprehensive Approach: A Narrative Review

**DOI:** 10.14336/AD.2023.0929

**Published:** 2024-08-01

**Authors:** Maria Tarnas, Andrzej Marszałek, Joanna Kufel-Grabowska, Sławomir Marszałek, Dariusz Wieliński, Jacek Zieliński

**Affiliations:** ^1^Poznan University of Physical Education, Department of Athletics, Strength and Conditioning, Poznan, Poland.; ^2^Poznan University of Medical Sciences and Greater Poland Cancer, Department of Oncologic Pathology and Prophylaxis, Poznan, Poland.; ^3^Medical University of Gdansk, Department of Oncology & Radiotherapy, Gdansk, Poland.; ^4^Poznan University of Medical Sciences, Department of Physiotherapy, Poland; Poznan University of Physical Education, Faculty of Physical Education in Gorzow Wielkopolski, Poland; Department of Oncologic Physiotherapy, Greater Poland Cancer Centre, Poznan, Poland.; ^5^Poznan University of Physical Education, Department of Anthropology and Biometry, Poznan, Poland.

**Keywords:** Pilates Method, cardiorespiratory function, diseases, health-related quality of life

## Abstract

Cardiorespiratory fitness (CRF) is established as a clinical vital sign in therapeutic strategy to restoring health of patients in medical conditions inclusive of age-related diseases. The beneficial effects of Pilates training (PT) are recognized for various aspects of health and fitness, but limited data present an impact on cardiorespiratory fitness. Thus, the current narrative review discusses the impact of the PT interventions on indicators of cardiorespiratory function among different patient groups to identify the mechanisms linking CRF with PT. The authors searched systematically databases: PubMed, Web of Science from inception to March 2023 and analyzed available data including finally 20 papers. In description of the findings PEDro Scale and final score was used. Analyzed data indicated: a) pleiotropic input of PT on improving physical performance in medical conditions; b) specific parameters characterizing effectiveness of PT in each group of patients according of disease; c) different range of static significance and effect size especially for such following indicators as: VO_2_ at VT (ml•kg^-1^•min^-1^), VO_2 peak/max_ (ml•kg^-1^•min^-1^), HR at VT (beats•min^-1^), HR_max_ (beats•min^-1^), VE (L•min^-1^). We also formulate and discuss potential physiological mechanisms of PT affecting CRF. This paper showed PT: a) has positive impact on broad spectrum of indicators of cardiorespiratory function by pleiotropic action among different patients’ groups; b) significant ameliorates quality of life that may contribute to long-standing behavior change of patients related with overall physical activity.

## 1. INTRODUCTION

According to the statement of the American College of Sports Medicine (ACSM) and the conceptions “health-related fitness” (H-RF) and “health-related quality of life” (HRQOL), cardiorespiratory fitness (CRF) is the fundamental component of health and well-being [1, 2]. Strong evidences confirm that a higher level of CRF is connected with a lower risk of cardiovascular morbidity and mortality as well as all-cause mortality. High CRF reflects a better capacity of the neuro-musculoskeletal system and metabolic energetic system. Published studies identify the positive relationships between CRF and health-related quality of life [3], mental health [4], cognitive functions [5]. Additional, high level of CRF could counteract symptoms of psychological problems causing by long-lasting stressors such as stress-related exhaustion, depression, anxiety, and sleep disturbances [6], which often accompany various diseases. It is worth to note that CRF is resulted from to opposite factors: sedentary lifestyle and physical activity [7]. Its reduction can be on one hand an effect of the abovementioned indicators and on the other it can be also impaired by clinical conditions (weakness, diseases process, side effects of treatment). What is more, CRF declines during aging and maintaining its relevant level guarantees independence and healthy aging [8]. American Heart Association proposed recommendations of CRF values for general fitness as well as a clinical vital sign in many diseases [9]. According to general reduction of physical activity in modern societies, additionally exacerbated by pandemic period and lockdown, preventive actions to increase CRF is a public health priority [10].

In the last two decades, numerous studies described the beneficial effects of Pilates training (PT) on various aspects of health and fitness [11, 12]. Such observations were recorded not only in groups with varying levels of physical fitness, age but also with specific disorders [8, 12, 13]. Although limited scientific data explain the effectiveness of PT, this method is applied as a component of treatment physical activity strategy in many diseases. PT was created by Joseph Hubertus Pilates in the beginning of the 20th century. The philosophy of PT is based on conception “body and mind”. It is a comprehensive system of exercise performed on the mat and specialized equipment and bases on six principles: centering, concentration, control, precision, flow and breathing [14, 15]. Applying these principles in training integrates the nervous, myofascial and skeletal systems and provides a better execution of each exercise leading to obtaining more effective results. PT includes dynamic, resistance exercises generating both isotonic (concentric, eccentric) and isometric contractions, accompanied by breathing, emphasizing the neuromuscular and fascial stimulation [16]. While PT is not considered as typical cardiorespiratory training, published studies suggest its positive effect on CRF [17, 18].

In the light of the high clinical relevance of CRF we systematically reviewed available databases to better understand the mechanisms linking CRF with PT. The current paper is narrative review of the literature included: i) the analysis of the intervention protocols used in practice research; ii) the overview of most commonly used parameters for evaluation of CRF; iii) the description of changes and their significance in the examined diagnostic parameters in specific groups of patients: metabolic, cardiovascular, respiratory, musculoskeletal and connective tissue diseases, cancers. Thus, this review investigated the impact of PT on CRF in the specific medical conditions and systematized available data.

## 2. MATERIAL AND METHODS

We focus in this narrative review on data as complex interactions between PT and cardiorespiratory fitness in specific medical conditions. The main aim of this study was to define potential exercise - intervention protocols which were applied in this topic. We compared the effects of differentiated applied protocols of PT including different intensity, time duration, frequency, and type of movement. What is more we evaluated if the induced changes were depended on group of subjects participated in analyzed studies.

### 2.1 Search Strategy.

The electronic database PubMed, Web of Science were searched from the inception to March 2023. The search algorithm was conducted using the following strategy: types of studies, participants, interventions, the medical specific conditions, and outcome assessment. Therefore, we used the free-terms/key words and matching synonyms of the following related words: (1) population: adults or “middle aged” or “young adults” or “young”; (2) intervention: “pilates” or “pilates method” or “pilates mat exercises” or “pilates machine exercises”; (3) outcomes: “cardiorespiratory fitness” or “aerobic capacity” or “peak oxygen uptake” or “maximal oxygen uptake” or “heart rate” or “blood pressure” or “ventilator functions” or “pulmonary functions” or” functional capacity” or “physical performance”; (4) the medical specific conditions: “metabolic diseases” or “cardiovascular diseases” or “respiratory/lung diseases” or “musculoskeletal and connective tissue diseases” or “cancers” or “obesity” or “diabetes” or “dyslipidemias” or “hypertensive” or “COPD” or “rheumatoid arthritis” or “osteoporosis” or “Parkinson’s diseases”.

### 2.2 Eligibility Criteria.

Publications meeting the following criteria were taken for final analysis: (1) provided information regarding the effects of Pilates interventions on cardiorespiratory fitness, the investigation of the cardiorespiratory features was a primary aim or coexisting; (2) were carried out as a randomized controlled trial (RCT), a controlled trial (CT), a quasi-experimental (Q-E); (3) all papers written in English and available full text; there was no limitation for publication year; (4) limited age from 10 years of age or older in the specific medical conditions, regardless of gender, diagnosed with metabolic, cardiovascular, respiratory, musculoskeletal and connective tissue, nervous diseases and cancers; (5) interventions classified as Pilates (on the mat, on the machines or both); (6) period of intervention was not shorter than 8 weeks. Studies were excluded if described the following points were included: (1) pregnant women, aging process, the sample included only healthy populations; (2) the study was a review, a case report or an abstract from a congress, a study protocol, a scientific reports, a practical examples; (3) outcome measurements were not described by parameters characterizing cardiovascular fitness; (4) the study used single training; (5) they were not written in English.

### 2.3 Study selection.

**Figure 1.**
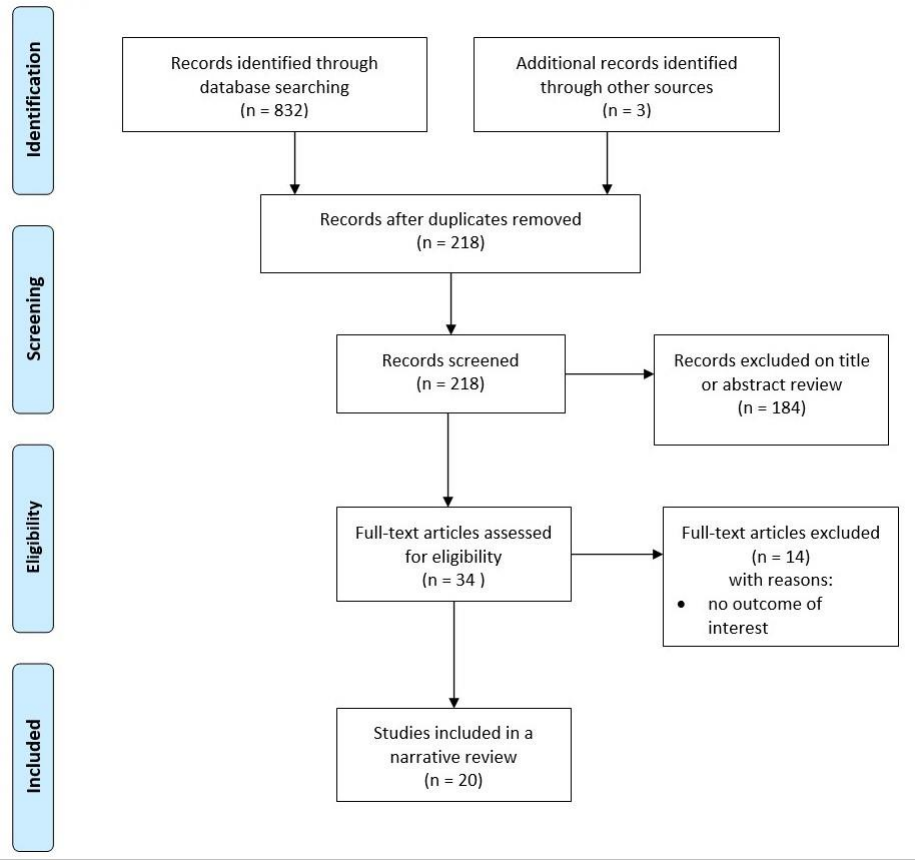
Flow diagram for the identification, screening, eligibility, and inclusion of studies.

Two authors (MT, JZ) identified records through database searching, performed data collection and extraction. Duplicate articles were removed. A total of 218 records were obtained. The titles and abstracts of potentially relevant articles were analyzed and for the purpose of this review 34 studies primarily selected. Due to a lack of consistency 14 papers were excluded. Thus, a total number of 20 papers were finally selected. All included studies were conducted in the last 13 years between, e.g., 2010 and 2023 with half of them (10 of 20) with the last 5 years. Details of the evaluation and selection process of the items are shown in Figure 1.

### 2.4 Included studies.

All publications included in the current study and their outcomes are summarized in Table 1. The studies were grouped according to the International Statistical Classification of Diseases and Related Health Problems ICD-11. The following disease groups have been recognized: metabolic [19-23], cardiovascular [24-27], respiratory [28, 29], musculoskeletal and connective tissue [30-33], nervous system [34-37], cancers [38]. In fifteen papers RCTs was implemented [19-21, 23-25, 27, 30-35, 38], in two non-RCTs [22, 26], and in three pre-post study comparison [28, 36, 37]. Additionally, the design of applied protocols was emphasized.

**Table 1:** The included studies.

Diseases	Authors/ Study design	Participants characteristics			Characteristic of PM intervention			
		Patients/ Recruitment spot	Age range/ Mean age	Sample size (% Female)	Frequency/ Period, sessions/ Missed trainings number	Certified Pilates teachers/ *Other specialists during training	Type/ *Accessories	Level of performance/ Numbers of sets and repetitions/ Progress of workload
Metabolic	Buttelli et al., 2021 CT	dyslipidemia nd	60-68 PG = 64.20 CG = 64.16	n = 26 (100%) PG = 20 CG = 6	45-50′/2-4xwk 10wks nd	nd	mat *not used	yes yes yes
	Jung et al., 2020 RCT	obesity nd	34-60 NPTG = 43.8 ± 8.6 HPTG = 47.2 ± 6.4 CG = 51.6 ± 6.5	n = 32 (100%) NPTG = 10 HPTG = 12 CG = 10	50′/3xwk 12wks, 36x nd	nd	mat *RB	yes 2 min. nd
	Wong et al., 2020 RCT	obesity around community	19-27 PG = 22 ± 1 CG = 23 ± 1	n = 28 (100%) PG = 14 CG = 14	60′/3xwk 12wks, 36x nd	nd	mat *nd	adapted protocol 1 sets 6-10 repetitions yes
	Rayes et al., 2019 RCT	overweight/ obesity school community	30-66 PG = 55.9 ± 6.6 CG = 45.5 ± 9.3	n = 60 (85%) PG = 22 AG = 21 CG = 17	60′/3xwk 8wks, 24x 3 (10%)	yes	mat + machines *nd	yes yes yes
	Tunar et al., 2012 RCT	T1DM hospitals	12-17 PG = 14.2 ± 2.2 CG = 14.3 ± 1.8	n = 31 (52%) PG = 17 CG = 14	45′/3xwk 12wks, 36x nd	yes *clinician	mat *nd	nd 3 sets 6-10 repetitions nd
Cardiovascular	Santos et al., 2020 CT	hypertensive Pilates studio	nd PG = 52.2 (6.5) CG = 49.5 (6.6)	n = 45 (100%) H-G = 30 N-G = 15	60’/2xwk 12wks, 24x nd	yes	machines *not used	nd yes yes
	Lim et al., 2017 RCT	chronic stroke rehabiliton center	nd PG = 63.2 ± 7.9 CG = 62.1 ± 6.7	n = 20 (45%) PG = 10 CG = 10	60’/3xwk 8wks, 24x nd	*2 specialists	mat *RB, B, MC	yes nd nd
	Martins-Meneses et al., 2015 RCT	hypertensive public advertisement	nd PG = 51.8 ± 4.3 CG = 49.0 ± 7.5	n = 44 (100%) PG = 22 CG = 22	60’/2xwk 16wks, 32x 8 (25%)	nd	mat *nd	nd 5-10 repetitions nd
	Guimaraes et al., 2012 RCPT	heart failure cardiology hospital	nd PG = 46 ± 12 CRG = 44 ± 11	n = 16 (31%) PG = 8 CRG = 8	60′/2xwk 16wks, 32x nd	yes *cardiac rehabilitation therapist	mat *RB, B, MC wobble board, rollers	nd nd nd
Respiratory	Hagag et al., 2019 RCT	COPD hospital	40-50 PG = 43.05 ± 2.07 CG = 42.09 ± 3.08	n = 38 (0%) PG = 19 CG =19	60’/3xwk 12wks, 36x nd	nd	mat *nd	yes yes nd
	Franco et al., 2014 Q-E	cystic fibrosis hospital	7-33 13.7 ± 7.4	n = 19 (63%)	60’/1xwk 16wks, 16x nd	nd	mat (individual session) * RB, B	nd nd nd
Musculoskeletal and connective tissue	Azab et al., 2022 RCT	JIA hospital	10-14 PG = 12.32 ± 1.67 CG = 11.56 ± 0.46	n = 37 (70%) PG = 19 CG = 18	25’/ 3xwk 12wks, 36x nd	nd *pediatric physiotherapist	mat * RB, B	nd yes nd
	Yentür et al., 2020 RCT	rheumatoid arthritis nd	18-65 PG = 48.20 ± 9.54 AG = 50.70 ± 10.66 P + AG = 51.90 ± 11.52	n = 30 (nd%) PG = 10 AG = 10 P + AG = 10	45’/2xwk 8wks, 16x nd	nd	mat * RB, B	yes nd nd
	Angın et al., 2015 RCT	postmenopausal osteoporosis rehabilitation center	40-69 PG = 58.23 ± 5.46 CG = 55.95 ± 9.22	n = 41 (100%) PG = 22 CG = 19	60’/3xwk 24wks, 72x nd	*physiotherapist	mat * RB, B	yes nd yes
	Küçükçakır et al., 2013 RCT	postmenopausal osteoporosis nd	45-65 PG = 56.6± 5.5 CG = 56.3± 5.0	n = 60 (100%) PG = 30 CG = 30	60’/2xwk 1 years nd	nd	mat * RB, B	nd nd nd
Nervous system	Abasıyanık et al., 2020 RCT	multiple sclerosis university hospital	nd PG = 42.5± 6.8 CG = 48.2 ± 11.79	n = 28 (85%) PG = 16 CG = 30	nd/1xwks 8wks nd	yes *physiotherapist	mat * RB, B	nd nd nd
	Cancela et al., 2018 Q-E	Parkinson’s diseases The Association of PD	52-80 69.13 ± 8.23	n = 16 (37%)	nd/2xwks 12wks nd	yes	mat * RB, chair gymnastic poles	yes yes nd
	Kalron et al., 2016 RCT	multiple sclerosis rehabilitation center	nd PG = 42.9 ± 7.2 CG = 44.3 ± 6.6	n = 45 (64%) PG = 22 CG = 23	30’ with 15‘ home exercises 12wks 2 (16%)	yes	mat *nd	nd nd nd
	Johnson et al., 2013 Q-E	Parkinson’s diseases The Neuro-Muscular Research Institute	54-77 67.6 ± 8.9	n = 10 (30%)	nd/1xwks 6wks nd	yes	mat + machine *B, steps	nd nd nd
Cancers	Eyigor et al., 2010 RCT	breast cancer university and oncology hospital	nd PG = 48,5 ± 7,6 CG = 49,7 ± 8,7	n = 52 (100%) PG = 27 CG = 25	60’/3xwk 8wks, 24x nd	yes *physiotherapist	mat *nd	yes yes nd

**CT**: controlled trial; **RCT**: randomized controlled trial; **RCPT**: randomized controlled pilot trial; **Q-E**: quasi-experimental; **T1DM**: type 1 diabetes mellitus; **COPD**: chronic obstructive pulmonary disease; **JIA**: juvenile idiopathic arthritis; **PG**: Pilates group; **CG**: control group; **AG**: aerobic group; **NPTG**: normoxic Pilates training group; **HPTG**: hypoxic Pilates training group; **P + AG**: Pilates + aerobic group; **H-G**: hypertensive group; **N-G**: normotensive group; **CRG**: cardiac rehabilitation group; **nd**: not determined; **RB**: resistance band; **MC**: magic circle; **B**: ball.

### 2.5 Evaluation of the validity of the included studies.

The PEDro methodological assessment scale was applied to evaluate the quality of the included studies [39]. The PEDro scale is a valid measure of clinical trials. Studies with a score between 4-6 were considered medium-quality studies and 7-10 high-quality studies (Supplementary Table 1).

### 2.6 Studied population.

Subjects participating in PT intervention were at different statuses of pathology of the disease, therapeutic regiments, and level of physical activity. Age characteristics were as follows appropriate in each disease group: metabolic (age range 12-68; mean age PG=41.2±4.1; CG=39.7±3.7) cardiovascular (age range was not determined; mean age PG=53.5±7.7; CG=51.2±8.0), respiratory (age range 7-50; mean age PG=28.4±4.7; CG=42.1±3.1); musculoskeletal and connective tissue (age range 10-69; mean age PG=43.9±5.5; CG=43.9±6.8); nervous diseases (age range in all samples was not determined; mean age PG=53.4±8.8; CG=57.8±11.5) and cancers (age range was not determined; mean age PG=48.5±7.6; CG=49.7±8.7). In each disease group females accounted majority and percentage was equal respectively for metabolic 86%; cardiovascular 83%, respiratory 21%, among musculoskeletal and connective tissue number was not determined, 91%; nervous diseases 63% and cancers 100%. Finally, 678 subjects took part in published studies, among them 390 belonged to Pilates groups (58%), 247 to control groups (36%) and 41 (6%) as comparison group underwent different types of training. Only one paper did not publish inclusion criteria. Patients were recruited in hospital and medical centers (12 of 20 studies), patient associations (1 of 20 study), around community (2 of 20 studies) and schools (1 of 20 study). The place of recruitment was not determined in 4 papers. The range of sample sizes (SS) and group sizes (Pilates group PG, control group CG, comparison group cG) were as follows: metabolic (SS n=26-60; PG n=17-22, CG n=6-17, cG n=21); cardiovascular (SS n=16-45, PG n=8-45, CG n=8-22, cG n=0), respiratory (SS n=19-38, PG n=19, CG n=19, cG n=0), musculoskeletal and connective tissue(SS n=30-60, PG n=10-30, CG n=10-30, cG n=10); nervous diseases (SS n=10-45, PG n=10-22, CG n=23-30, cG n=0) and cancers (SS n=52, PG=27, CG n=25, cG n=0).

### 2.7 Protocols of interventions of PT.

The reviewed Pilates interventions consisted of training sessions whose frequency was between 1 and 4 per week. It turned out that 9 out 20 interventions had 3 sessions per week. The interventions ranged from 8 weeks to 1 year. From the group of 20 papers it turned out that 16 interventions lasted from 8 to 12 weeks, with 7 planned for 12 weeks. The duration of a single session was planned 60' (11 studies), 50' (1 study), 45' (3 studies), 30' (1 study), 25’ (1 study). The time duration of a single session was not determined in 3 publications. Only 3 protocols indicate the permitted numbers of missed training ranged from 2 (16%) to 8 (25%) [19, 25, 34]. Among the 20 studies, 17 were based on mat Pilates exercises. Two studies combined both type of Pilates exercise on the mat and machine [19, 37]. Machines were only used in 1 intervention [26]. In 11 interventions there were used the several types of equipment to attain various goals: an elastic band, swiss balls, small balls, chairs, gymnastic poles, magic circles [23, 24, 27, 28, 30-33, 35-37]. In 9 articles the information of the certification and experience of Pilates teachers was provided [19, 20, 24, 26, 34, 35-38]. In 6 interventions PM exercise were performed under supervision by medical specialists and physiotherapists [24, 27, 30, 33, 35, 38]. Any information regarding leaders of interventionists was presented in 8 publications. The detailed description of the training program: the names of exercise and load (number of series, repetitions, and weekly progression) were published in 3 articles [19, 21, 22]. The exercise intensity was monitored during each training session using: a) the subjective method Borg Scale [25, 29]; b) the objective external load control by a heart rate (HR) [19, 24]. In one study [23], effects of PT in normoxic condition were compared with PT in hypoxic conditions.

### 2.8 Indicators used in the evaluation of the effectiveness of applied protocols PM training.

Selected parameters of CRF were evaluated to establish an impact of PT. In the group of patients with metabolic diseases especially parameters of physical performance oxygen uptake at ventilatory threshold (VO_2VT_), oxygen uptake at respiratory compensation point (VO_2RCP_), peak/maximal oxygen uptake (VO_2peak/max_), power and efficiency of cardiovascular system (heart rate at ventilatory threshold HR_VT_, heart rate at respiratory compensation point HR_RCP_, rest heart rate HR_rest_, maximal heart rate HR_max_, systolic diastolic blood pressure BP_s,d_, pulse pressure PP, mean blood pressure MBP, mean arterial pressure MAP, augmentation index Alx, augmented pressure AP, brachial/ankle wave velocity baBWV) were recorded. Most of the presented results have shown statistically significant improvement of the abovementioned parameters. The range of effect size for obtained results was: 0.33-0.81 for VO_2VT_, VO_2RCP_, VO_2peak/max_ respectively; for HR_VT_, HR_RCP_, HR_max_ 0.48-0.90; -0.10-0.12 for BP_s,d_ in normoxic conditions; -0.48-0.65 for BP_s,d_ in hypoxic conditions; -0.26-(-0.04) for PP; -0.03 for MAP in normoxic conditions; -0.60 for MAP in hypoxic conditions; -0.04 for baBWV in normoxic conditions; -0.39 for baBWV in hypoxic conditions. Similar parameters were also used among cardiovascular group of patients. Physical performance was expressed in values of absolute or relative VO_2peak/max_. Efficiency of cardiovascular system was examined by HR_rest_, HR_VT_, HR_RCP_, HR_max_, HRpost training. HR average value based on 3 measurements just post awake, from day, before asleep, oxygen pulse (Pulse O_2_), double product (DB), blood pressure (SD, DB, MBP) and values of creatine kinase activity (CK). The range of effect size for obtained results wasn't defined in cardiovascular group of patients. Among patients with respiratory diseases, it was demonstrated that PT had beneficial effects on CRF what was mainly characterized by changing parameters of efficiency of respiratory system minute ventilation (VE), forced 1-second expiratory volume (FEV_1_), forced vital capacity (FVC), maximum inspiratory pressure (MIP), maximum expiratory pressure (MEP). Most of them have achieved statistically significant effects. The effect size for these findings wasn't indicated. Among patients with dysfunctions of the musculoskeletal system and connective tissue, one study presented an improvement of VO_2peak/max_, HR_max_, VE. These results have shown statistically significant improvement, and the effect size was determined for them in the range of 0.81-0.90. In this group of patients, the 6-minute walk test (6MWT) functional test was used to assess CRF indicating statistically significant improvement and effect size 0.24. However, in nervous diseases group of patients, the respiratory parameters (MIP, MEP) and functional capacity were evaluated 6MWT or the 2-minute walk test (2MWT), the 2-minute step test (2minST), the 5-meter walk test (5-mW). Presented results have shown statistically significant improvement. The effect size was only determined for 2minST test and was -0.93. Only one study revealed the impact of PT on CRF among breast cancer patients and obtained outcomes indicated a positive, statistically significant effect but with no indication of the effect size. Diverse methods were used in order to assess CRF: directly method using gas analyzer [19, 24, 27, 30], indirect such as 6MWT or 2MWT, 2minST, 5-mW [29, 31-38], or the regression equation was used for estimated parameters [23]. Although, statement published by the AHA pointed VO_2_ peak and minute ventilation/carbon dioxide production (V_E_/VCO_2_) slope parameters as the “gold standard” in assessment exercise capacity, in many groups of patients these indicators were not determined [40].

## 3. RESULTS

Selected parameters characterizing the cardiovascular fitness were classified according to rules described in Table 2. The results are presented by determining the impact of PT on the CRF and additionally the significance of induced changes was considered.

**Table 2:** The measured indicators of cardiorespiratory function in studies with PM intervention in specific groups of diseases.

	Groups of diseases											
	Metabolic		Cardiovascular		Respiratory		Musculoskeletal and connective tissue		Nervous system		Cancers	
Measured indicators	Trend	ES range	Trend	ES range	Trend	ES range	Trend	ES range	Trend	ES range	Trend	ES range
**Physical performance indicators**												
**VO_2_ at VT** (ml·kg^-1^·min^-1^)	↑^*^ [19]	0.60	-	-	-	-	-	-	-	-	-	-
**VO_2_ at RCP** (ml·kg^-1^·min^-1^)	↑^*^ [19]	0.48	-	-	-	-	-	-	-	-	-	-
**VO_2_ max** (ml·min^-1^)	-	-	↑^*^ [27]	nd	-	-	-	-	-	-	-	-
**VO_2_ peak/max (ml·kg^-1^·min^-1^)**	↑^*^ [19] ↔ _n_ [23] ↑ _h_^*^ [23]	0.33 [19] 0.01 _n_ [23] 0.34 _h_ [23]	↑^*^ [24, 27]	nd [24, 27]	-	-	↑^*^ [30]	0.81	-	-	-	-
**Power** (W)	↑_peak_^*^ [20] ↑_mean_^*^ [20]	nd	-	-	-	-	-	-	-	-	-	-
**RER**	-	-	↔ [24]	nd	-	-	-	-	-	-	-	-
**Circulatory efficiency indicators**												
**Pulse O_2_** (mL/beats·min^-1^)	-	-	↑ [24]	nd	-	-	-	-	-	-	-	-
**# HR** (beats·min^-1^)	-	-	↑ [25]	nd	-	-	-	-	-	-	-	-
**† HR** (beats·min^-1^)	-	-	↓ _N_ [26] ↓ _H_ [26]	nd	-	-	-	-	-	-	-	-
**HR rest** (beats·min^-1^)	-	-	↓ [24] ↓ _N_^*^ [26] ↓ _H_^*^ [26] ↓^*^ [27]	nd [24, 26, 27]	-	-	-	-	-	-	-	-
**HR at VT** (beats·min^-1^)	↑^*^ [19]	0.90	-	-	-	-	-	-	-	-	-	-
**HR at RCP** (beats·min^-1^)	↑^*^ [19]	0.56	-	-	-	-	-	-	-	-	-	-
**HR max** (beats·min^-1^)	↑^*^ [19]	0.48	↑ [24]	nd	-	-	↑^*^ [30]	0.84	-	-	-	-
**DB** (beats·min^-1^·mm Hg)	-	-	↓_#_ [25]	nd	-	-	-	-	-	-	-	-
**SBP** (mm Hg)	↓^*^ [21] ↑ _n_ [23] ↓ _h_ [23]	nd [21] 0.12 _n_ [23] -0.48 _h_ [23]	↓_rest_ ↑_max_ [24] ↓_#_^*^ [25] ↓_rest/N_ [26] ↓_rest/H_ [26]	nd [24-26]	-	-	-	-	-	-	-	-
**DBP** (mm Hg)	↓^*^ [21] ↓ _n_ [23] ↓ _h_ [23]	nd [21] -0.10 _n_ [23] -0.65 _h_ [23]	↓_rest_ ↑_max_ [24] ↓_#_^*^ [25] ↓ _rest/N_ [26] ↓ _rest/H_ [26]	nd [24-26]	-	-	-	-	-	-	-	
**PP** (mm Hg)	↑ [21] ↑_n_ [23] ↔ _h_ [23]	nd [21] 0.26 _n_ [23] -0.04 _h_ [23]	-	-	-	-	-	-	-	-	-	-
**MBP** (mm Hg)	-	-	↓_#_^*^ [25]	nd	-	-	-	-	-	-	-	-
**MAP** (mm Hg)	↓^*^ [21] ↔ _n_ [23] ↓ _h_ [23]	nd [21] - 0.03 _n_ [23] - 0.60 _h_ [23]	-	-	-	-	-	-	-	-	-	-
**Alx** (%)	↓^*^ [21]	nd	-	-	-	-	-	-	-	-	-	-
**AP** (mm Hg)	↓^*^ [21]	nd	-	-	-	-	-	-	-	-	-	-
**baPWV** (cm/s)	↔ _n_ [23] ↓ _h_ [23]	-0.04 _n_ [23] -0.39 _h_ [23]	-	-	-	-	-	-	-	-	-	-
**CK** (U/L)	-	-	↓ _N_ [26] ↓ _H_^*^ [26]	nd	-	-	-	-	-	-	-	-
**Respiratory efficiency indicators**												
**VE** (L·min^-1^)	-	-	-	-	-	-	↑^*^ [30]	0.90	-	-	-	-
**FEV _1_** (% of predicted)	-	-	-	-	↑_F_ ↑_M_ [28] ↑^*^ [29]	nd [28, 29]	-	-	-	-	-	-
**FVC** (% of predicted)	-	-	-	-	↑_F_ ↓ [28] ↑^*^ [29]	nd [28, 29]	-	-	-	-	-	-
**MIP** (cm H_2_O)	-	-	-	-	↑_M_^*^ ↑_F_^*^ [28]	nd	-	-	↑^*^ [35]	nd	-	-
**MEP** (cm H_2_O)	-	-	-	-	↑_M_ ↑_F_^*^ [28]	nd	-	-	↑^*^ [35]	nd	-	-
**Functional tests indicators**												
**6MWT(m)**	↑^*^ [22]	1.41	-	-	↑^*^ [29]	nd	↑^*^ [31-33]	nd [31, 32] 0.24 [33]	↑^*^ [34, 35]	nd [34, 35]	↑^*^ [38]	nd
**2MWT (m)**	-		-	-	-	-	-	-	↑^*^ [34]	nd	-	-
**2minST**	-	-	-	-	-	-	-	-	↑^*^ [36]	-0.93	-	-
**5-mW (m)**	-	-	-	-	-	-	-	-	↑^*^ [37]	nd	-	-

**List of abbreviations**: **ES**: effect size; **VO**: minute oxygen uptake; **VT**: ventilatory threshold; **RCP**: respiratory compensation point; VO_2_ **peak/max**: peak/maximum oxygen uptake; **RER**: respiratory exchange ratio; **PulseO_2_**: oxygen pulse; **HR**: heart rate; **HR rest**: rest heart rate; **HR max**: max heart rate; **#**: average value based on 3 measurements just post awake, from day, before asleep; **†**: post training; **DB**: double product HRxBP; **SBP**: systolic blood pressure; **DBP**: diastolic blood pressure; **PP**: pulse pressure; **MBP**: mean blood pressure; **MAP**: mean arterial pressure; **Alx**: augmentation index; **AP**: augmented pressure; **baPWV**: brachial/ankle wave velocity; **CK**: creatine kinase activity; **VE**: minute ventilation; **FEV_1_**: forced 1-second expiratory volume; **FVC**: forced vital capacity; **MIP**: maximum inspiratory pressure; **MEP**: maximum expiratory pressure; **6MWT**: 6-minute walk test; **2MWT**: 2-minute walk test; **2minST**: 2-minute step test; **5-mW**: 5-meter walk test; ↓ decrease; ↑ increase; ↔ no changes; ^*^ statically significant; _n_ normoxic; _h_ hypoxic; _N_ normotensive; _H_ hypertensive; **nd**: not determined.

**Figure 2.**
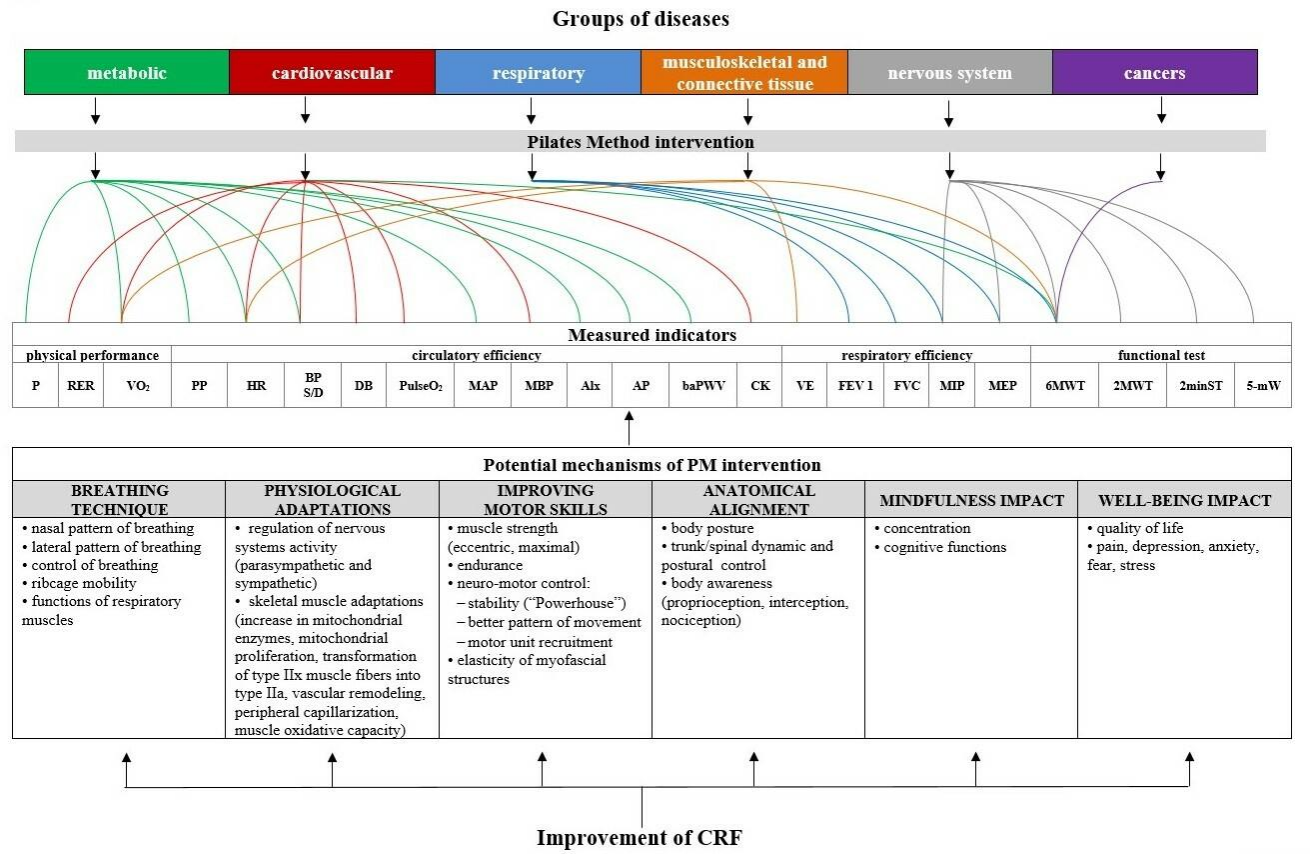
Potential physiological mechanisms of PT affecting the improvement of CRF.

Obtained data includes changing of divers measured indicators in participants with different spectrum of diseases: metabolic, cardiovascular, respiratory, musculoskeletal, and connective tissue, nervous system, cancers (Figure 2). Emerging evidence highlighted that PT was the alternative and complementary type of physical activity which finally contributed to the improvement of the CRF. In the analyzed results we propose some possible mechanisms underlying Pilates-induced changes which are also presented in Figure 2.

Among the 20 selected studies, 17 were assessed using the PEDro scale. The RCT, RCPT, CT design studies were qualified for analysis. The score ranged from 4 to 8 points. The 14 included studies (70%) demonstrated medium methodological quality with scores falling within the range of 4 to 6 points. The 6 studies obtained 4 points, 5 obtained 5 points and 3 of them 6 points. The 3 included studies (15%) exhibited high-quality methodology (2 of them obtain 7 points and 1 gets 8 points).

## 4. NARRATIVE COMMENTS

Based on the broad spectrum of PT effects obtained among different patient groups, it is worth formulating some potential mechanisms of the impact on CRF. Finally, these mechanisms lead to induce pleiotropic action improving physical performance in specific medical conditions as well as a significant amelioration quality of life.

The first crucial mechanism relates to a breathing technique because PT assumes: i) a more efficient pattern of the breathing by lateral/interocostal breathing; ii) restoration of nasal breathing and iii) prolonged an exhalation and an inhalation phase. Using the lateral breathing pattern provides effective, safe, and stable core functions of the trunk. It is a result of abdominal muscles maintaining their tension during inhale as exhale phase. Lateral breathing also improves chest musculoskeletal functions by the ribcage mobility and enhancement of respiratory muscles [20, 28, 41]. PT properly activate intercostals muscles, diaphragm and synchronize its functions with other core muscles (pelvic floor muscles, anterior and posterior abdominal muscles) [42-44]. Restoring physiological activation of diaphragm modifies ventilatory functions, cardiac output through an improvement of splanchnic vascular bed [24, 26, 45, 46], regulation of stroke volume, heart rate variability (HRV) and balance in the sympathetic and parasympathetic activation [45, 47]. In turn restoring the physiological nasal breathing pattern corrects and positions properly the tongue in the palate (back and downward) of which disturbances are often observed during breathing through the mouth. The right position of the tongue provides to a properly alignment of head eliminating an unfavorable strain on the cervical joints and the deep muscles that stabilize the spine, reintroducing their functional postural support role [48]. The improvement nose patterns have also the immunological meaning due to warming inspired air, increasing its humidity, filtration toxicants- mucociliary clearance and producing mucus and secrete immunoglobulins. Interesting biochemical and physiological aspect of nasal breathing is producing nitric oxide (NO) [49, 50], what is related to cardiovascular functions including arterial oxygenation, better capillarization and stimulating angiogenesis. PT in therapeutic approach assumes breathing control by slow and deep breathing frequency near 0.1 Hz (6 breaths per minute). This technique initiates neurophysiological processes associated with a shift away from sympathetic dominance toward a net increase in parasympathetic (vagal) tone. Control of breathing reduces the risk of hyperventilation what has a meaning not only respiratory function but also for metabolic processes [47]. Induced changes ameliorate fatigue tolerance and extended time to exhaustion. Therefore the ability of slow breathing technique has been promised in a variety of cardiorespiratory and stress-related disorders [51].

The second mechanism indicates the potential physiological adaptations associated with performing PT breathing technique and effects of resistance training. PT breathing regulates a) the activity of the parasympathetic and sympathetic nervous systems; b) biomechanical functions in the body cavities (thoracic and abdominopelvic). Its beneficial effects on respiratory, cardiovascular, circulatory functions. The indicators and their directions of change are presented comprehensively in the results section. PT could be also considered as resistance training characterized by low to moderate intensity. It can be not ruled out that PT as resistance training could stimulate physiological adaptations: an increase activity of mitochondrial enzymes, mitochondrial proliferation, transformation of type IIx muscle fibers into type IIa, and vascular remodeling [52]. Still, further research is needed to confirm this effect induced by PM. Also, muscle conditioning is related to improvement peripheral capillarization and muscle oxidative capacity, what may have a positive effect on VO_2_ at the ventilatory threshold (VT) [19]. These adaptations might be visible among patients with lower physical condition.

The third potential mechanism assumes an improving of motor skills including neuro-motor control, myofascial flexibility [20, 53], and muscle endurance and strength [19, 54]. These beneficial changes can be obtained by better recruitment of motor units, properly flow movements, efficient movement patterns and better stability of body (“Powerhouse”) [8, 57]. Both, concentric and eccentric types of contraction during Pilates exercise play important role for abovementioned neural-muscular adaptation changes [56, 57]. Pilates exercises initiated by stabilization of core musculature provide to control movements of limbs and optimal range of motion [29, 58]. Additionally, PT-induced changes contribute to reduction of overload in peripheral joints. Therefore, restoring an adequate level of central body stabilization in patients promotes improved mobility, better locomotion, and capacity to perform everyday activities.

The fourth potential mechanism concerns the positive impact of PT on the anatomical alignment of body, control of trunk and spine in static and dynamic conditions [54, 59-61], and kinesthetic body awareness [24], which is the result of a proper stimulation and transmission of signals between proprioreceptors, nociceptors, interoreceptors and analyzing centers of the nervous system. All together factors ensure the optimal biomechanical each of body segments function [62] which prevents the impairment of postural stability particularly among patients with chronic lung diseases and obstructive lung disease [29]. It is worth noting the role of connective tissue which intermediates in the transmission of forces, and it is significant source of senso-motor information. Its function is integrated with the nervous system (central and peripheral). Given such a large impact on connective tissue PM is considered and propsed as fascial training.

The fifth area of PT impact is related to body& mind principles expressed in mindfulness - concentration, movement control and its precision, which finally promote the improvement of cognitive functions [35, 63]. Mindfulness is related to the teaching process applied in PT, which includes specific verbal instructions, "hands on" technique in order to correct, movement support and maintain a positive mental attitude during training. This approach helps exercising people to attend to the sensory attention to bring awareness to bodily processes such as breathing, sensing, and initiating movement from the core. It is worth to note that keeping patient’s positive mental attitude focuses their attention on small incremental progress rather than emphasizing limitations [64]. Improved self-efficacy through increasing body awareness and somatic education is essential from a clinical point of view and strategy of the management treatment [65]. Overall, PM is considered as a form of somatic education which improves process of psychophysical integration [64].

The last mechanism presents a beneficial influence of PT on the well-being and improvement of quality of life [36, 38, 66], which follow from emotional variability connected with anxiety, fear, stress and even chronic pain [31, 33, 55, 67]. All these abovementioned disruptions are significant barriers to engage in training programs. This aspect is particularly important regarding emotional connection with somatic response. Still, the underlying mechanism is very difficult to prove because both these areas (emotional and somatic) are assessed separately. The issue of interception explains links of visceral interoceptive disorders triggered by stress and negative emotions and indicates potential ways to alleviate the consequences of psychosomatic disorders. Sensations from proprioceptors are usually received by the sensorimotor cortex, and signals from interoreceptors are processed by an insular within the cerebral cortex. Changes in the processing of fascial interoceptive information are suspected to be followed by feelings such as anxiety and depression. These changes are combined with an increased dose of interoceptive information in the form of so-called "noise". In addition, this processing is enhanced by the course of the disease or the negative aspect of beliefs relating to oneself. To effectively stimulate interoceptive sensation, attunement methods should be used during therapeutic activities. They promote inner mindfulness directed at feeling, for example, skin sensations, general sensations or warmth, feelings of inner tranquility. Therefore, mindfulness-based therapies are observed to have a positive effect on the improvement of illness that is associated with stress, anxiety, or depression. Perceptions look for the grounding of processes in the musculoskeletal system, remote fascial, sympathetic, visceral-somatic, or even endocrine connections. Other significant barrier to engage in training programs is the co-occurrence of high risk of bodily injury, falls and physical limitations in clinical conditions like Parkinson's Disease. PT provides widespread opportunities to use in disorders by apply adaptation of exercises to the level of physical fitness, taking into account motor skills, body awareness and cognitive functions; safe starting positions (vertical, horizontal, with adaptation of positioning of appropriate points and planes of support); varied exercise accessories.

In summary, the conducted researches have some limitations and analysis of the published results is complicated by following factors: i) the heterogeneity of samples as well as age range of participants, predominant participation of women, as well as homogeneity of the medications used by patients in disease states; ii) methodological differences in studies design and differences in applied protocols (intensity, number of repetitions and rest time between sets, lack of monitoring of the training intensity using direct methods and load, mats or apparatus, sessions per week and program duration, a lack of information about the instructors' certification); iii) problem of methodological quality (non-blinded studies, small samples, number of drop-out above 15%, lack of randomization between control and experimental groups; iiii) a lack of follow up and determine long-term effects of PT post 6 months or 1 year training. From a clinical point of view these limitations complicate formulating precise conclusions and recommendations.

## 5. PRACTICAL APPLIATION AND FURTHER RESEARCH PERSPECTIVE

Even though, the previous investigations [17] observed that Pilates exercises are effective in improving CRF. The current study: a) have proposed a set of evaluation criteria for CRF parameters to ensure consistent assessment depending on the evaluated patient dysfunction and comparison of future research; b) have explained potentially mechanisms lead to induce pleiotropic action PT improving physical performance in specific medical conditions; c) have identified a problem of methodological quality in clinical trial that essential for future studies.

Results from this review emphasize the clinical utility of PT and present this method as an effective type of training in improving cardiovascular fitness in specific medical conditions. PT can be a feasibility method in clinical practice. Mat Pilates exercises do not require large financial investments. PT is becoming a valued method used by physiotherapists. Moreover, PT is a repetitive training (exercise program) what professionals in clinical practice can reproduce and at the same time teach patients to apply the exercises at home. Adaptation of Pilates exercises to clinical limitations and needs can be a safe perspective of training in varied medical departments as well as in COVID patients.

Future direction of research should include: i) the study designs providing of high methodological quality of clinical trials allowing repeatability of studies and conclusion; ii) the search for simple, indirect tools to measure the intensity of PT exercises and their effects; iii) the inclusion of blood indicators measuring inflammation reduction; iiii) define some specific tests which lead to assess physical performance before and post PT application. In the interdisciplinary model of patient care, it is important to study the quality of life of patients because considers all aspects of the patient's life and developing long-standing behavior change. This assessment is an important tool for assessing readiness, potential and priorities in physical activity. Moreover, clinicians emphasize that assessing quality of life can be a measure of the effectiveness of physical activity programs.

## Supplementary Materials

The Supplementary data can be found online at: www.aginganddisease.org/EN/10.14336/AD.2023.0929.


