# Supplementary material for: Effects of Pilates Training on Cardiorespiratory Functions in Medical Conditions - Comprehensive Approach: A Narrative Review
Source: Aging Dis. 2024 Aug 1;15(4):1771–83. doi: 10.14336/AD.2023.0929 (PMC11272188; doi:10.14336/AD.2023.0929)
Supplement: Supplementary file 1 [file AD-15-4-1771-s.pdf]

SUPPLEMENTARY DATA

Effects of Pilates Training on Cardiorespiratory Functions in Medical Conditions - Comprehensive Approach: A Narrative Review

Maria Tarnas, Andrzej Marszałek, Joanna Kufel-Grabowska, Sławomir Marszałek, Dariusz Wieliński, Jacek Zieliński

© 2023.Tarnas M. et al. Published online at http://www.aginganddisease.org/EN/10.14336/AD.2023.0929

SUPPLEMENTARY DATA

Supplementary Table 1. PEDro scale and PEDro score of included studies.

Diseases	Authors		PEDro Scale Item										
		Eligibility criteria (items does not score)	Random allocation	Concealed allocation	Baseline comparability	Blind subjects	Blind therapists	Blind assessors	Adequate follow-up	Intention -to-treat analysis	Between-group comparisons	Point estimates and variability	PEDro final score
Metabolic	Buttelli et al., 2021	no	yes	no	yes	no	no	no	no	no	yes	yes	4/10
	Jung et al., 2020	no	yes	no	yes	no	no	no	yes	no	yes	yes	5/10
	Wong et al., 2020	yes	yes	no	yes	no	no	yes	yes	no	yes	yes	6/10
	Rayes et al., 2019	yes	yes	no	no	no	no	yes	no	no	yes	yes	4/10
	Tunar et al., 2012	no	yes	no	yes	no	no	no	yes	no	yes	yes	5/10
Cardiovascular	Santos et al., 2020	yes	no	no	yes	no	no	no	no	yes	yes	yes	4/10
	Lim et al., 2017	yes	yes	yes	no	no	no	no	yes	yes	yes	no	5/10
	Martins-Meneses et al., 2015	yes	yes	no	yes	no	no	no	no	no	yes	yes	4/10
	Guimaraes et al., 2012	yes	yes	no	yes	no	no	no	no	no	yes	yes	4/10
Respiratory	Hagag et al., 2019	yes	yes	yes	yes	no	no	yes	yes	yes	yes	no	7/10
	Franco et al., 2014	Q-E not qualified for analysis											
Musculoskeletal and connective tissue	Azab et al., 2022	yes	yes	yes	yes	no	no	yes	yes	yes	yes	yes	8/10
	Yentür et al., 2020	yes	yes	no	yes	no	no	no	yes	yes	yes	yes	6/10
	Angın et al., 2015	yes	yes	no	yes	no	no	no	yes	no	yes	yes	5/10
	Küçükçakır et al., 2013	yes	yes	no	yes	no	no	no	yes	yes	yes	yes	6/10
Nervous system	Abasıyanık et al., 2020	yes	yes	no	yes	no	no	no	no	yes	yes	yes	5/10
	Cancela et al., 2018	Q-E not qualified for analysis											
	Kalron et al., 2016	yes	yes	yes	yes	no	no	yes	yes	no	yes	yes	7/10
	Johnson et al., 2013	Q-E not qualified for analysis											
Cancers	Eyigor et al., 2010	yes	yes	no	yes	no	no	no	no	no	yes	yes	4/10

© 2023.Tarnas M. et al. Published online at http://www.aginganddisease.org/EN/10.14336/AD.2023.0929
